# The political economy of sugar-sweetened beverage taxation: an analysis from seven countries in sub-Saharan Africa

**DOI:** 10.1080/16549716.2021.1909267

**Published:** 2021-04-20

**Authors:** Anne Marie Thow, Safura Abdool Karim, Mulenga M. Mukanu, Gemma Ahaibwe, Milka Wanjohi, Lebogang Gaogane, Hans Justus Amukugo, Charles Mulindabigwi Ruhara, Twalib Ngoma, Gershim Asiki, Agnes Erzse, Karen Hofman

**Affiliations:** a Menzies Centre for Health Policy, School of Public Health, Charles Perkins Centre, The University of Sydney, Sydney, Australia; b SAMRC/Centre for Health Economics and Decision Science - Priority Cost Effective Lessons for Systems Strengthening (PRICELESS SA), Faculty of Health Sciences, School of Public Health, University of the Witwatersrand, Johannesburg, South Africa; c Health Policy and Management Unit, University of Zambia School of Public Health, Lusaka, Zambia; d Economic Policy Research Centre (EPRC), Makerere University, Kampala, Uganda; e Health and Systems for Health Unit, Nairobi, Kenya; f Department of Health Promotion & Education, Boitekanelo College, Gaborone, Botswana; g Community Health Department, School of Nursing, Faculty of Health Sciences, University of Namibia, Windhoek, Namibia; h School of Economics, University of Rwanda, Butare, Rwanda; i Economic Social Research Foundation (ESFR), Muhimbili University of Health and Allied Sciences, Dar es Salaam, Tanzania

**Keywords:** Noncommunicable disease, tax, sugar-sweetened beverage, political economy, policy

## Abstract

**Background**: Non-communicable diseases are on the rise across sub-Saharan Africa. The region has become a targeted growth market for sugar-sweetened beverages, which are associated with weight gain, cardiovascular diseases and diabetes.

**Objective**: To identify politico-economic factors relevant to nutrition-related fiscal policies, and to draw lessons regarding strategies to strengthen sugar-sweetened beverages taxation in the region and globally.

**Methods**: We collected documentary data on policy content, stakeholders and corporate political activity from seven countries in east and southern Africa augmented by qualitative interviews in Botswana, Namibia, Kenya and Zambia, and stakeholder consultations in Rwanda, Tanzania and Uganda. Data were analysed using a political economy framework, focusing on ideas, institutions, interests and power, and a ‘bricolage’ approach was employed to identify strategies for future action.

**Results**: Non-communicable diseases were recognised as a priority in all countries. Kenya, Zambia, Rwanda, Tanzania and Uganda had taxes on non-alcoholic beverages, which varied in rate and tax base, but appeared to be motivated by revenue rather than health concerns. Botswana and Namibia indicated intention to adopt sugar-sweetened beverage taxes. Health-oriented sugar-sweetened beverage taxation faced challenges from entrenched economic policy paradigms for industry-led economic growth and was actively opposed by sugar-sweetened beverage-related industries. Strategies identified to support stronger sugar-sweetened beverage taxation included shifting the economic discourse to strengthen health considerations, developing positive public opinion, forging links with the agriculture sector for shared benefit, and leadership by a central government agency.

**Conclusions**: There are opportunities for more strategic public health engagement with the economic sector to foster strong nutrition-related fiscal policy for non-communicable disease prevention in the region.

## Background

Nutrition-related non-communicable diseases (NCDs), including diabetes, cardiovascular diseases (CVDs) and some forms of cancer, are a major cause of death and disability globally. NCDs caused a third of all deaths in sub-Saharan Africa (SSA) in 2016, up from 28% in 2010 [1], with approximately 60% of NCD deaths in the region due to CVDs [2]. Obesity, which is a major driver of NCDs, is on the rise. The proportion of the adult population that is overweight or obese in the African region has increased from 28.4% in 2000 to 41.7% in 2016 [2]. Women in the African region are twice as likely to be overweight or obese as males, and since 2013, more CVD related deaths were observed among women than men [2,3]. This significant gender differential in NCD risk and mortality has remained almost constant for decades.

The economic cost of NCDs in the African region is considerable with the cost of CVD alone including medical, non- medical and productivity costs estimated at US$6 billion in 2010. The cumulative direct and disability costs of diabetes in middle income countries globally ranged from US$17 – 61 billion in 2010 [4]. The costs continue to escalate as dietary risk factors for NCDs increase across the region, in line with the well–documented dietary transition from traditional to ‘modern’ and more processed diets [5–7]. Fruit and vegetable consumption is declining [8], with similtaneous increased consumption of unhealthy, cheap processed foods high in salt, fat and sugar [9]. These processed foods include sugar-sweetened beverages (SSBs), which have no nutritional value and the consumption of which is associated with weight gain, CVD and diabetes [10,11].

Addressing increased SSB consumption has emerged as a recent health policy concern in the region. Population growth, expanding economies and a growing middle-class mean that SSA is an emerging global market for SSBs, and these countries offer new opportunities for multinational companies seeking to expand their consumer base [12,13]. To further economic growth, governments are actively supporting industry investment and expansion, including through increasing regional integration. The 2019 African Continental Free Trade Area agreement was designed to foster intra-African trade, to support cross-continental industries and value chains, and to promote economic growth [14]. Livelihoods versus wellbeing is a critical debate that must be incorporated into decision making in order to address the real costs of a growing obesity epidemic in emerging economies across the continent where children are still stunted and undernourished.

The multinational food and beverage industry is increasingly targeting the SSA market through strategies to grow market size and share, expansion through purchasing local companies, and direct investment. The leading sugary beverages companies on the continent are Pepsi Co and Coca-Cola, although they are often present as subsidiaries of their multinational parent companies. Pepsi Co has been able to ‘tap into’ the regional market through a recent acquisition of Pioneer Foods, which has 22 food and beverage brands that are exported to 80 countries, including many in the region [13]. Multinational food companies actively position themselves as fostering economic growth in SSA, with a focus on supporting vulnerable food producers. For example, the Coca-Cola Company is partnering with small-scale fruit farmers in order to enable them to participate in local markets and supply chains [15]. As these companies expand and their sales grow, concerns are being raised by the public health community about the impact on the increasing prevalence of NCDs and on future economic growth and development [16].

While SSA governments are pro-active in recognising the burgeoning NCD epidemic, there is limited evidence of the use of strong regulatory approaches [12]. Taxes on SSBs have been recommended by the World Health Organization (WHO) as part of a package of comprehensive measures to prevent and control NCDs [17]. Evidence to date indicates that well designed taxes can reduce consumption at a population level, with concomitant health benefits [18,19]. While mostly proven to work across the globe, SSB taxes have proved controversial and challenging to implement at rates associated with health benefits, in many cases due to industry opposition [20]. Concerns such as regressivity in terms of price (a disproportionate impact on the poor) and impacts on industry and employment while frequently raised are not an issue [20,21]. There is significant scope for strengthening taxation at the national level across SSA; but progress is likely to be hampered by politico-economic barriers [20,22]. This paper examines the political economy of SSB taxation in seven SSA countries in east and southern Africa. The aim of this analysis is to increase understanding of the politico-economic dynamics relevant to nutrition–related fiscal policies, and to draw lessons regarding strategies to strengthen the design and implementation of SSB taxes for NCD prevention for the African region and globally.

## Methods

### Study design and theoretical frameworks

This is part of a broader study which analysed the policy landscape related to NCD and SSB taxation policies in seven SSA countries [23]. Policy analyses were conducted in Botswana, Kenya, Namibia, Rwanda, Tanzania, Uganda and Zambia, focussed on policy content, context, process and actors for SSB taxation and informed by political science theories [23–28]. These countries have emerging NCD epidemics, and represent a spectrum of different levels of existing taxation that applies to SSB.

This paper presents a secondary political economy analysis of the data collected by the research teams in each country, in line with the primary research question for this regional study: *What are key factors influencing policy decisions regarding SSB taxation, and what strategies could strengthen taxation for nutrition-related NCD prevention?*

Political economy analysis has been recommended for studies related to the ‘how’ of nutrition policy, due to its explicit recognition of the roles of politics, economics, and institutions in shaping policy decisions [29]. This study drew on Campbell’s institutionalist approach to political economy analysis, which emphasises the importance of understanding *ideas and paradigms* that underlie policy decisions, the *institutional context* in which decisions are made, and *stakeholder interests and power* [30]. ‘Ideas’ include underlying (and sometimes taken for granted) assumptions and perceptions, as well as concepts and theories, that are evident in policy debates, and are sometimes called policy paradigms. In particular, theories of policy making suggest that understandings and perceptions of the nature of the ‘policy problem’ (in this case, nutrition-related NCDs) are important influences on the policy solutions that are seen as appropriate. ‘Institutions’ are norms and structures; they include what is generally thought of as formal institutions (e.g. government agencies) as well as formal and informal rules and procedures. Stakeholder ‘interests’ refer to the objectives and goals of different actors, and the indicators they see as important. ‘Power’ refers to which actors are influencing policy making, and mechanisms through which this occurs; power can be exercised overtly or at a paradigmatic level, through shaping cultural norms [31,32].

### Data collection

Documentary data (policy documents, reports and media) were collected in all seven countries between October 2018 and April 2019. We extracted data on policy content, stakeholder interests and influence, and corporate political activity for each country using a standard matrix to ensure systematic extraction of data relevant to pre-determined themes, which included stakeholder interests, policy frames (particularly in relation to the nature of the ‘policy problem’ and ‘policy solution’, and mechanisms for actor influence. The documentary data were verified through consultations with policy stakeholders in all countries. Detailed methods are described elsewhere [23].

Additional data on policy paradigms and frames, stakeholder interests and industry activity were collected through qualitative interviews with policy stakeholders in Botswana (n = 6), Kenya (n = 10), Namibia (n = 13) and Zambia (n = 10) [25,26,28]. Interviewee selection was informed by the documentary stakeholder analysis. Interviews were semi-structured, and based on Kingdon’s multiple streams approach, with questions asked about perceptions of the ‘policy problem’ of NCDs as well as ‘policy solutions’ and the political context. The interviews were transcribed in full, and data were coded and analysed by the research team in each country. All researchers conducting interviews were granted approval from the relevant research ethics bodies (University of Namibia Research Ethics Committee (clearance number SOPH/434/2018); Amref Health Africa – Ethical and Scientific Review Committee (Kenya), Ethics number: P593/2019; and Zambia ERES Converge IRB (IRB No.00005948, EWA No. 00011697), approval number 2018-Nov-021, and all participants provided informed consent.

### Data analysis

This paper presents a secondary political economy analysis of the data collected by the research teams in each country. The documentary data from all seven countries and findings from the interview data from four countries were analysed based on pre-determined themes, informed by the political economy frameworks underpinning this regional study: ideas, institutions, stakeholders interests, and power [30]. The country-level study findings were coded to these themes using a matrix (country/theme) by the lead researcher, and then analysed across countries to identify key factors influencing policy decisions, and to determine strategies that could strengthen SSB taxation for NCD prevention, with input from the research teams in each country. These coded data were then used to inform a process of ‘bricolage’, an approach in which strategies were identified to build upon and subvert existing ideas and institutional structures in ways that would promote stronger action (namely in relation to SSB taxation) for NCD prevention [30]. All authors input into the analytical process; the pre-determined themes for data extraction were developed collaboratively, and co-authors discussed and provided commentary on the results throughout the secondary analysis.

The results are structured in line with the analytical approach. We first present an overview of the relevant policy context in all seven countries, to describe the actions that governments are taking and the evident policy responsibilities for NCD prevention. We then present the findings of the political economy analysis, focusing on ideas, institutions, stakeholder interests and power. Finally, we present the opportunities for strategic health sector engagement to strengthen SSB taxation that arose from the analysis.

## Results

### Overview of the policy context relevant to SSB taxation

Five of the countries had excise taxes on non-alcoholic beverages (soft drinks) in place: Zambia, Uganda, Kenya, Tanzania and Rwanda (Table 1). Zambia was the only country that presently has a differential SSB tax, of 3% on imported beverages and 0.5% on local drink products [28]. In general the taxes appeared to be largely motivated by the need to raise revenue for government and were often applied to ‘luxury goods’ more broadly, with SSBs part of this category. However, there were health links made in three of the five countries with an excise tax. The tax in Zambia was proposed following Ministry of Health lobbying, and was in part justified on health grounds [28]. In Kenya, there was an additional tax in place on sugar confectionery, which was reported to be based on both health and revenue concerns [26, 33] [25] (Table 1). In Uganda, an explicit health link was made by earmarking excise revenue from sugar, soft drinks and other products; 2% of the levy was for the HIV/AIDS Trust Fund [24]. The governments of Botswana and Namibia currently have no excise tax, but were both considering SSB taxation, explicitly as part of their NCD prevention strategies (Table 1).

**Table 1.** Overview of consideration relevant to SSB taxation, nutrition and NCDs in food-related policies

Policy	Botswana	Kenya	Namibia	Rwanda	Tanzania	Uganda	Zambia
**whole-of-government Policies**							
National Development Plan	Mentions the need to address NCDs and their associated lifestyle risk factors and promotion of healthy lifestyle ^a^	Mentions need to address food security ^b^	Mentions multisectoral approach to prevention and control of NCDs and agricultural production for improved nutrition ^c^	Mentions malnutrition as hindering productivity. Supports alleviation of the burden of NCDs and their risk factors ^d^	Mentions multi-sectoral action on NCDs ^e^	Mentions promotion of healthy lifestyles for NCD prevention ^f^	Mentions efforts to reduce NCDs through health promotion and implementing programmes to promote good nutrition ^g^
Vision	State of the Nation Address 2018, the President mentioned that government was considering options to reduce consumption of unhealthy products such as SSBs ^h^	Mentions food and nutrition security ^i^	Mentions creating access to abundant, hygienic, and healthy food based on food security ^j^	Mentions food security and NCDs as key to development	Mentions general policy commitment to NCDs ^k^	Mentions NCD prevention through healthy lifestyles at household and community level and preventative health system^l^	Mentions strengthening nutrition care practices for those affected by NCDs ^m^
**Health Sector Specific**							
National Health Policy	Mentions restricting marketing of unhealthy foods and SSBs and introducing fiscal measures to promote healthy diet and discourage unhealthy food ^n^	Mentions NCD prevention through addressing major risk factors – including unhealthy diets ^o^	Mentions general priorities for public health, which include nutrition and NCDs ^p^	Mentions promoting dietary diversity as a way to address overnutrition ^q^	Mentions malnutrition and obesity with lack of physical activity identified as a risk factor ^r^	Mentions NCDs as a health and nutrition related problem addressed through knowledge and information ^s^	Mentions NCD prevention and public policies to promote health to enhance quality of life of the population ^t^
National Health Sector Strategic Plan	Mentions developing new guidelines to improve access to healthy food and increasing awareness of NCDs. Also mentions utilising ‘sin taxes’ to generate revenue ^u^	Mentions halting and reversing the rising burden of NCDs ^v^	Mentions prevention and management of NCDs ^w^	Mentions coordination of stakeholders in nutrition programs and production of healthy food crops. Mentions increasing knowledge of good nutrition practices and strengthening intersectoral collaboration for NCDs prevention ^x^	Mentions multi sectoral approaches to addressing preventable causes of disease but not specifically NCDs. Mentions nutrition in first 1000 days^y^	Mentions provision of NCD prevention and control services: and community awareness of nutritious foods ^z^	Mentions embedding nutrition interventions in plan to prevent NCDs. Also commits to multisectoral action to prevent and control NCDs ^aa^
National Food and Nutrition Policy	*Not identified*	Mentions adequate nutrition for optimum health and general food production to increase access for food security ^bb^	Focusses on food and nutrition security. Mentions nutrition transition and aims to improve quality of nutrition including through smallholder, local production ^cc^	Strategic directions on all forms of malnutrition, to increase knowledge around diet-related NCDs and improve household food security ^dd^	*Not identified*	Acknowledges NCDs require multisector approach ^ee^	Mentions need to address challenges of malnutrition ^ff^
NCD Strategic Plan	Mentions multisectoral approach to prevention and control of NCDs, specifically mentions creation of a legislative and policy environment conducive to healthy living and provision of subsidies for healthy foods ^gg^	Mentions implementation of health-related legislation including limiting salt, saturated and trans fatty acids and sugar content of processed foods and beverages ^hh^	Mentions multisectoral approach to minimise duplication and increase coordination ^ii^	Mentions that NCD prevention and control should be integrated into existing healthcare system ^jj^	Mentions increases in NCD burden as attributable to western fast foods, level of physical activity, alcohol and tobacco usage ^kk^	*No policy in place at point of field work*	Mentions introducing a multisectoral plan and NCD task force to address NCD prevention. Specifically mentions addressing the limitations of industry self-regulation ^ll^
**Education sector specific**							
Schools Policy	*Not able to obtain*	Mentions increasing awareness and intake of adequate, locally available and nutritious foods among school children and their communities ^mm^	Mentions access to diverse nutritious food in schools; Scale up school feeding programme; provision of balanced and fortified meals; nutrition education ^nn^	Mentions integration of NCD prevention and control in school curricula at various levels of education. Recommends research support to generate evidence for monitoring NCDs risk factors control and NCD policy-making ^oo^	Mentions promoting positive behaviour change for prevention NCDs by promotion of healthy life styles and the importance of healthy eating and physical fitness	Mentions that schools may include a variety of foods in the menu to ensure diet adequacy and diversity, with emphasis on fruits and vegetables. ^pp^	Mentions plans to improve nutrition status of learners and initiating health promoting activities 43
**Agriculture sector specific**							
National Agriculture policy	*Not included*	*Not included*	Mentions food quality, security, self-sufficiency and soveriegnty ^qq^	Mentions food and nutrition security through production, accessibility and affordability of healthy foods ^rr^	Policy promotes increasing local sugarcane production to meet SSB industry needs ^ss^	Mentions promotion of production and consumptions of nutritious foods, including indigenous foods, at the household level ^tt^	Mentions addressing food insecurity ^uu^
**Trade and Economic**							
Fiscal policy		Excise tax of KES 10 (USD 0.10) per litre on all non- alcoholic beverages, including bottled water and excise tax on sugar confectionary. (at KES 20 (USD 0.20) per kg) ^vv^	Mentions VAT zero-ratings or exemptions for some goods and services, such as staple foods ^ww^	Excise tax of 39% excise tax on soft drinks, including non-SSBs, with revenue earmarked broadly for government ^xx^	Excise tax of TZS 54 (USD 0.02) per litre on non-alcoholic beverages and levy on imports for revenue earmarked broadly for government ^yy^	Excise tax of UGX 200 (USD 0.05) per litre on non-alcoholic beverages and levy on sugar with revenue earmarked for HIV/AIDS Trust Fund ^zz^	Excise tax of ZMW0 .3 (USD 0.02) per litre on non-alcoholic beverages for with revenue earmarked broadly for government ^aaa^
National Trade and related Policy	Mentions restrictions on importing, exporting and licensing of agricultural products to ensure food security ^bbb^	*Not included*	Foreign Investment Act 1995 includes support for food and beverage companies ^ccc^	Ensure compliance of traded goods with regulations and that they match ‘the needs of the entire society’ ^ddd^		Mentions that growth in trade should support food security, and the importance of compliance with standards that protect health ^eee^	

Blank cells indicate that the research team did not identify specific policy content relevant to SSB taxation, nutrition and NCDs in the policy document; ‘not included’ indicates that this sector was not included in the country-level study. The summaries of the policy documents presented in the table reflect the best endeavours of the research team to identify and obtain the relevant national policies during the period of data collection, and to accurately extract the relevant information.

Sources:

aRepublic of Botswana. *National Development Plan 11 April 2017– March 2023*. Botswana: Republic of Botswana, 2016.

bMinistry of Health. *The Constitution of Kenya 2010*. Nairobi: Government of Kenya, 2010.

cGovernment of Namibia. *Namibia’s 5th National Development Plan (NDP5)*. Namibia, 2017.

dRepublic of Rwanda. *7 Year Government Programme: National Strategy for Transformation (NST 1) 2017 – 2024*. Rwanda, 2017.

eMinistry of Finance and Planning. *National Five Year Development Plan 2016/17 – 2020/21*. Tanzania: The United Republic of Tanzania, 2016.

fThe Republic of Uganda. *Second National Development Plan 2015/16– 2019/20 (NDPII)*. Kampala, Uganda: Republic of Uganda, 2015.

gMinistry of National Development and Planning. *Seventh National Development Plan 2017– 2021*. Lusaka, Zambia, 2017.

hDr. Mokgweetsi E.K. Massi, President of the Republic of Botswana. *State of the Nation Address*. Gaborone, Botswana: Republic of Botswana, 2018.

iGovernment of Kenya. *Kenya Vision 2030*. Nairobi: Government of Kenya, 2007.

jGovernment of Namibia, Office of the President. *Namibia Vision 2030: Policy Framework for Long-Term National Development*. Windhoek, Namibia: Government of Namibia, 2004.

kThe United Republic of Tanzania. *The Tanzania Development Vision 2025*. Tanzania: The United Republic of Tanzania, 2000.

lThe Republic of Uganda. *Vision 2040*. Kampala, Uganda: Government of Uganda, 2007.

mMinistry of Finance. Vision 2030: *A Prosperous Middle-Income Nation by 2030*. Zambia: Republic of Zambia, 2006.

nMinistry of Health. *National Health Policy ‘Towards a Healthier Botswana’*. Gaborone, Botswana: Republic of Botswana, 2011.

oMinistry of Health. *Kenya Health Policy 2012– 2030*. Nairobi, 2012.

pMinistry of Health and Social Services. *National Health Policy Framework 2010– 2020*. Namibia: Government of Namibia, 2010.

qMinistry of Health. *Health Sector Policy*. Kigali, Rwanda: Republic of Rwanda, 2015.

rMinistry of Health, Community Development, Gender, Elderly and Children. *The National Health Policy 2017*. Tanzania: The United Republic of Tanzania, 2017.

sMinistry of Health. *The Second National Health Policy*: Promoting People’s Health to Enhance Socio-economic Development. Uganda: The Republic of Uganda, 2015.

tMinistry of Health. *National Health Policy*. Lusaka, Zambia: Republic of Zambia, 2012.

uMinistry of Health. *Integrated Health Service Plan: A Strategy for Changing the Health Sector for Health Botswana 2010 – 2020*. Botswana: Government of Botswana, 2010.

vRepublic of Kenya. *Health Sector Strategic and Investment Plan (KHSSP) July 2013 – June 2017*. Nairobi, Kenya: Ministry of Medical Services and Ministry of Public Health and Sanitation, 2013.

wMinistry of Health and Social Services, Primary Health Care Directorate Family Health Division. *National Multisectoral Strategic Plan for Prevention and Control of Non-Communicable Diseases (NCDs) in vi 2017/18 – 2021/22*. Namibia: Government of Namibia, 2017.

xMinistry of Health. *Health Sector Policy*. Kigali, Rwanda: Republic of Rwanda, 2015.

yMinistry of Health and Social Welfare. *Health Sector Strategic Plan July 2015 – June 2020*. Tanzania: United Republic of Tanzania, 2015.

zMinistry of Health. *Health Sector Development Plan 2015/16– 2019/20*. Kampala, Uganda: The Republic of Uganda, 2015.

aaMinistry of Health. *Zambia National Health Strategic Plan 2017– 2021*. Zambia: Republic of Zambia, 2017.

bbMinistry of Public Health and Sanitation. National Nutrition Action Plan 2012– 2017. Nairobi: Government of Kenya, 2012

ccRepublic of Namibia, National Food security and nutrition council, *Food and Nutrition policy for Namibia*. Windhoek, Namibia; 1995

ddMinistry of Local Government, Ministry of Health and Ministry of Agriculture and Animal Resources. *National Food and Nutrition Strategic Plan*. Kigali, Rwanda: Republic of Rwanda, 2014.

eeMinistry of Agriculture, Animal Industry and Fisheries and Ministry of Health. *The National Food and Nutrition Strategy*. Uganda: The Republic of Uganda, 2005

ffNational Food and Nutrition Commission of Zambia. *National Food and Nutrition Strategic Plan for Zambia 2011– 2015*. Zambia: Republic of Zambia, 2011.

ggMinistry of Health & Wellness. *Botswana Multi-Sectoral Strategy for the Prevention and Control of Non-Communicable Diseases 2018– 2023*. Botswana: Republic of Botswana, 2018.

hhMinistry of Health. *Kenya National Strategy for the Prevention and Control of Non-Communicable Diseases 2015 – 2020*. Nairobi, 2015.

iiMinistry of Health and Social Services, Primary Health Care Directorate Family Health Division. *National Multisectoral Strategic Plan for Prevention and Control of Non-Communicable Diseases (NCDs) in Namibia 2017/18 – 2021/22*. Namibia: Government of Namibia, 2017

jjMinistry of Health. *Rwanda Noncommunicable Diseases National Strategic Plan July 2014– June 2019*. Kigali, Rwanda: Republic of Rwanda, 2014.

kkMinistry of Health, Community Development, Gender, Elderly and Children. *Strategic and Action Plan for the Prevention and Control of Non Communicable Diseases in Tanzania 2016– 2020*. Tanzania: Government of the United Republic of Tanzania, 2016.

llMinistry of Health. *Zambian Strategic Plan 2013 – 2016 Non–Communicable Diseases and their Risk Factors*. Lusaka, Zambia: Government of the Republic of Zambia, 2013.

mmMinistry of Education, Ministry of Health, Ministry of Agriculture, Livestock and Fisheries. *National School Meals and Nutrition Strategy* 2017–2022. Kenya: Republic of Kenya, 2017.

nnRepublic of Namibia; Ministry of Education, Arts and Culture, NAMIBIA SCHOOL: FEEDING POLICY; 2019

ooMinistry of Education. *Education Sector Policy*. Rwanda: Republic of Rwanda, 2014.

ppMinistry of Health & Ministry of Education and Sports. Uganda School Health Policy. The Republic of Uganda; 2008.

qqMinistry of Education. *National School Health and Nutrition Policy*. Lusaka, Zambia: Republic of Zambia, 2006.

rrRepublic of Namibia; *Namibia Agriculture policy*, 2015

ssMinistry of Agriculture and Animal Resources. *Strategic Plan for Agriculture Transformation 2018 – 24*. Rwanda: Republic of Rwanda, 2018.

ttMinistry of Agriculture, Food Security and Cooperatives. *National Agriculture Policy*. Tanzania: The United Republic of Tanzania, 2013.

uuMinistry of Agriculture, Animal Industry and Fisheries and Ministry of Health. *National Agriculture Policy*. Uganda: The Republic of Uganda, 2013.

vvMinistry of Agriculture and Ministry of Fisheries and Livestock. *Second National Agricultural Policy*. Lusaka, Zambia: Government of the Republic of Zambia, 2016.

wwGovernment of Kenya. *Kenya Gazette Supplement: National Assembly Bills 2018*. Nairobi, 2018.

xxRepublic of Namibia; *Fiscal policy and National Economy . Aligning Public Expenditure with the Medium-Term Development Plan for Socio-Economic Development*; 2015

yyMinistry of Trade and Industry. *Rwanda Trade Sector for Quality, 2010*. Rwanda: Republic of Rwanda, 2010.

zzzThe United Republic of Tanzania. *The Excise (Management and Tariff) Act*. Sect. 4 (2019).

aaaThe Republic of Uganda. *The Excise Duty (Amendment) (NO.2) Act, 2018*, (2018).

bbbGovernment of Zambia. *The Customs and Excise (Amendment) Act, 2018*, (2018).

cccUNCTAD. *Trade Policy Framework: Botswana*. New York and Geneva: UNCTAD, 2016.

dddGovernment of the Republic of Namibia. *Value-Added Tax Amendment Act*, Namibia: Government Gazette of the Republic of Namibia, 12 (2015).

eeeThe Republic of Uganda. *Uganda National Trade Policy 2007: Trading Out of Poverty Into Wealth and Prosperity*; 2007.

The broader policy context related to NCD prevention and treatment included comprehensive action by the health sector in all countries, which was the primarily locus of policy responsibility for NCDs (Table 1). All countries identified NCDs as a priority in their National Health Policies or Strategic Plans, and six countries has NCD-specific policy documents. There were also specific school-based initiatives in collaboration with the education sector identified in Kenya, Rwanda, Tanzania and Zambia (Table 1). However, the documentary analysis also indicated limited consideration of NCDs in agricultural and economic/industrialisation policy priorities related to economic growth and employment in all countries (Table 1). These focussed generally on increasing industry activity and productivity.

This analysis of policy content formed the basis for the political economy analysis, by summarising the key policy priorities and dynamics relevant to SSB taxation and diet-related NCDs across sectors (Table 1). In particular, the policy content identified a key point of policy tension between SSB taxation, which can act as a constraint on industry activity, and economic sector objectives towards private sector led growth. The findings below detail the underlying political economy of these policy tensions, and are organised thematically using Campbell’s institutionalist approach [30].

### Ideas

The framing and paradigms evident in the documentary analysis and interviews indicated that underlying assumptions regarding the relative importance of preventing NCDs and the contribution of the beverage industry to economic growth hampered adoption of strong SSB taxation.

With respect to perceptions of the ‘policy problem’ relevant to SSB taxation, NCDs were recognised by governments and stakeholders in all countries as a significant problem and a ‘whole-of-government’ priority (Table 1). In all countries except Kenya, NCDs were noted as a challenge in the National Development Plan (or equivalent document). In the Kenya Vision 2030, NCDs are not specifically mentioned as a problem, but acknowledged indirectly through a health-related objective to improve preventive health services [34]. Overall the critical health priorities emphasised in health policy documents were treatment (particularly for communicable diseases) and ongoing concern about undernutrition rather than NCDs. In Botswana, Kenya and Namibia, previous success in using taxation of tobacco seemed to raise the profile of NCD prevention as a policy issue. Discussion of proposed SSB taxation referred to successful use of fiscal policy to address tobacco consumption as a major NCD risk factor.

The dominant paradigm evident in policy documents outside of the health sector was the imperative of economic development, in the context of persistent poverty and the need to promote food security. Industry actors (including the food industry) were framed as important to achieve economic growth and maintain employment opportunities, and there was high level support for industry-led growth in National Development Plans and other economic policy documents in all countries. For example, in Zambia, the agriculture and manufacturing sectors were identified as key in reaching economic growth objectives [28]. In Tanzania and Uganda, the Government had an explicit priority regarding sugar industry growth. In Uganda, the explicit objective of the National Sugar Policy (2010) is to promote and sustain steady industrial growth and development and improve the competitiveness of the sugar sector [24]. In Tanzania, the National Agriculture Policy encourages the increase of sugar cane production to meet SSB industry needs and alleviate poverty.

Stakeholder interviews in Zambia, Namibia and Kenya indicated public sentiments regarding dietary transition contrasted traditional diets (as healthy) with modern diets (as unhealthy). Concerns centred both on a shift away from traditional staples such as maize and millets towards more refined staples such as rice, and on increased consumption of fast foods and highly processed foods. The latter concern was evident in policy documents. For example, the Kenya Food and Nutrition Security Policy explicitly identified ‘imports and local production of more processed foods’ as a cause of NCDs.

### Institutions

In all countries, there was recognition that new structures were necessary to co-ordinate a multisectoral approach, and multisectoral strategy documents had been developed to address NCDs to support these. In Uganda, Kenya, Tanzania, Rwanda and Zambia, NCDs were the responsibility of the Ministry of Health, which then convened multisectoral action on NCDs and nutrition. Four countries, Botswana, Namibia, Kenya and Zambia, had multisectoral committees or technical working groups for NCDs in place (or being established). Only in Namibia and Botswana were the multisectoral strategies and coordinating structures situated outside the Ministry of Health, as the responsibility of the central Government agency (the Office of the Prime Minister or the Presidency); notably, these were the two countries publicly considering the adoption of an SSB tax. Across all seven countries, limited engagement of the economic sectors of government in these multisectoral forums was reported.

Actors outside of government were also engaged in institutional structures and policy processes relevant to NCD prevention and SSB taxation. Civil society organizations (CSOs) and academic institutions in Kenya and Namibia, were named explicitly as role-players in NCD-related policies in the health sector, and thus included in formal government processes surrounding decision-making and implementation. There has been ongoing advocacy and lobbying by CSOs and academics for a tax on SSBs in Uganda and Kenya (i.e. a SSB-specific tax, reflecting health considerations, rather than the current beverage tax), and broadly for action on NCDs in Rwanda. In the other countries, there was very little civil society activity with respect to nutrition-related NCDs. Academics were identified as being engaged in relevant policy processes in Zambia, Kenya, Namibia and Uganda as advocates and sources of policy-relevant evidence. For example, academics in Zambia had presented evidence to the Government regarding the potential benefits of an SSB tax in 2018 [35]. Government policy documents in the economic sector note that industry is a key stakeholder. Our study identified the main institutional engagement by industry actors as direct lobbying of government, the primary purpose being to influence the discourse around the beverage industry to emphasise their economic contributions and not the health impacts.

### Stakeholder interests

SSB taxation involves a range of stakeholders within and outside of government, but especially the Ministries of Finance, Health and Agriculture, and the beverage industry. There was evident tension in the policy documents between the interests of different government sectors. Ministries of Health were strongly in favour of strong action for NCD prevention, and in Zambia, Botswana and Namibia this included recommendations for taxation on SSBs, reiterated by respondents in the health sector. However, we also found that taxation of SSBs was perceived as contrary to the prevailing economic paradigm in most countries; whereby industry interests aligned with government economic interests for employment and economic growth, and were given preference over public health interests. For example, respondents in Namibia noted that tax exemptions through The Foreign Investment Act of 1995 would likely increase supply, reduce costs and ultimately increase consumption of SSBs, contrary to Ministry of Health priorities. Similarly, media in Uganda and Tanzania showed that industry publicly lobbied against taxation (in Uganda reducing the tax and in Tanzania opposing increases) on the basis of the need to promote industry competitiveness.

In Namibia, Zambia, Uganda, Kenya and Rwanda, there was alignment between Agriculture and Health sector priorities, with production of healthy foods considered in Agricultural policies as part of food and nutrition security strategies. For example, the Uganda National Agriculture Policy included policy objectives on promoting production and consumption, at the household and community level, of nutritious and diverse foods, including indigenous foods. However, in other countries, such as Tanzania, the priorities for food security within the Ministry of Agriculture focussed on production, with little consideration of health and nutrition.

Civil society organizations and coalitions with an interest in NCDs had a strong presence in Uganda and Kenya, and had been advocating specifically for a tax on SSBs. In Rwanda and Tanzania, there was evidence of a broad coalition of NGOs with interest in NCDs. However, within Rwanda and Uganda in particular, the focus on Civil society organization lobbying regarding NCDs was primarily in relation to access to medicines, i.e. to treat rather than prevent NCDs.

The primary interest of SSB-related industry actors was evident in documentary analysis perceived by interviewees to be maximising profits, including through increasing their market share in all countries. In Namibia, Tanzania and Uganda, SSB industry actors were explicitly positioned against SSB taxation; this was based on their roles as ‘job providers’ and their contribution to the economy. The objective of their lobbying was to prevent any threats to SSB market growth. An industry strategy evident in Kenya, Rwanda and Zambia was publicization and promotion of their contribution to the economy through corporate social responsibility activities such as sports sponsorship.

The primary interest of academic actors in all countries was the generation of evidence to support action on SSBs and NCDs. In Kenya, Namibia and Uganda the study identified a need for further research to generate local evidence on the need for, and potential impacts of, SSB taxation. Respondents noted that evidence of the impact in other countries was not perceived as sufficiently transferable to inform local policy.

### Power

The two most influential stakeholders identified in the political and economic context were government and industry. Data from all countries highlighted the power held by government actors at sectoral level, through their ‘formal’ decision-making powers. For example, Ministers of Finance are key in making decisions on taxation policy, and Ministers of Health are lead decision-makers on NCD policy, although they have no formal remit or mandate for finance policy decisions. However, in Uganda, Namibia, Kenya and Tanzania, the lack of success in achieving taxes high enough for health impact that are specifically on SSBs (suggesting limited power within the health sector) was attributed to a perception by some policy makers that there was weak evidence for the link between SSBs and NCDs, and particularly a lack of local evidence for the potential effect of the tax.

The SSB industry was identified in all countries as a powerful policy actor, primarily due to the significant resources held by industry, as well as their deliberate positioning as pivotal to economic growth and development. There was indication that direct lobbying against SSB taxation was effective in all countries. In Uganda, industry lobbying resulted in a decrease in the tax rate applied to non-alcoholic beverages, and the tax on confectionary was removed due to concerns about competitiveness in the region. In Kenya and Tanzania, the failure to increase the SSB tax (as advocated for by the public health community) was attributed to industry lobbying. The cautious approach of the Government of Botswana to adopting a SSB tax was attributed to industry advocacy regarding the tax as a threat to job security. In Rwanda, the SSB industry was one of the largest tax payers in the country, and was identified as a potential source of influence in lobbying the Government to reduce the tax. In most countries the major multinational SSB companies had the largest market share and were identified as having highly sophisticated and influential tactics; in some cases the smaller ‘local’ companies were also subsidiaries or bottlers for multinational brands and thus also benefited from these tactics. For example, in Uganda the two largest SSB companies (Century Bottling and Crown Beverages) bottle Coca-Cola and Pepsi respectively. In countries where local companies were producing non-global branded SSBs, the local industry actors were also very influential, particularly where they were large employers or had a strong industry representative body. For example, a respondent in Kenya highlighted the role and influence of the Manufacturer associations in lobbying successfully to reduce SSB taxation.

The normalization of SSB consumption across all countries likely contributes to the ‘paradigmatic’ power exercised by industry actors: the more culturally accepted the product is, the less likely it is to be perceived as a cause for concern. Respondents and policy documents from the health sector in Zambia, Kenya, Uganda, Rwanda, Tanzania and Botswana identified rising consumption of SSBs, particularly among youth, as a public health concern, and noted aggressive advertising by the industry as a factor normalizing consumption.

### Strategies to strengthen SSB taxation

In this section, we draw on the secondary analysis conducted using a ‘bricolage’ approach, together with reflection on the international literature to outline three strategies to enhance adoption of stronger fiscal policy measures to address SSBs and NCDs.

#### Strategy 1: framing NCD prevention as a necessity for economic productivity and growth

First, the research identified an evident disconnect between the economic and the health policy sectors with respect to food industry activities. This points to a need to shift the economic discourse, and particularly discourse at a whole-of-government (e.g. National Development Plan) level, such that the societal and economic value of taking pro-health action on NCDs is recognised. The economic case for prevention of NCDs is strong, given the high health sector expenditure and lost productivity associated with NCD treatment [36] in a region that cannot afford either. Recent work in Fiji has indicated that framing NCD prevention as critical for economic growth can enable health policy makers to work within a (generally) neoliberal economic policy paradigm [37]. The development of new metrics for national development that support an understanding of nutrition and NCDs as a precursor to economic productivity will be pivotal to such an approach. The World Bank’s Human Capital Index is an example of positioning nutrition as critical to a productive future workforce [38]. These efforts to change frames and metrics may be most effective if they are supported by local research and evidence to counter industry arguments. The findings of this study suggest that local research on nutrition and NCDs can be more powerful than international evidence in informing policy making.

#### Strategy two: marketing healthy traditional diets and de-normalizing SSB consumption

Second, this research suggests that an important precursor to strengthening SSB-related taxation will be supporting positive public opinion regarding action on NCDs. The SSB industry appears to have been effective in normalizing consumption of SSBs in these SSA countries, particularly among youth. As such, one important strategy to shift public opinion will be de-normalizing SSB consumption through specific campaigns about the health harms. This was effective in Mexico and South Africa, where civil society and the health sector conducted extensive social marketing campaigns regarding the effects of SSB consumption on health, leading to widespread public acceptance of the SSB tax [39,40].

Intervention to de-normalize sugar consumption and SSB consumption in particular can be helpfully complemented by government intervention to promote healthier diets. In particular, through promoting healthy traditional foods, including marketing to influence prevailing perceptions regarding ‘ideal’ diets, as well as interventions to increase access to more convenient forms of traditional foods to reduce time need for food preparation. This is particularly important in contexts of transitions to urbanisation. Although this study highlighted that commonly consumed traditional foods are not always healthy (for example, processed maize meal in Zambia), indigenous diets across SSA were generally relatively healthy and diverse [41]. Investing in social marketing that celebrates and encourages traditional diets through strategies to increase access to, and convenience of, these foods can create a ‘demand side’ pull by increasing consumer awareness of the health benefits of traditional diets. This would help to create local agricultural markets and also support efforts to promote healthy, minimally processed diets. Multifaceted interventions that address cultural dimensions as well as access to traditional healthy foods have been effective in creating demand for healthy traditional foods, and supporting healthier diets in Korea [42,43] and Pohnpei (Federated States of Micronesia) [44,45].

#### Strategy three: promoting shared benefits between agriculture and health

Third, there is an opportunity for the health sector to work with the agriculture sector to promote shared benefits; in particular, the health and economic benefits of healthy food production, as well as the potential use of SSB tax revenue to promote this. This study revealed a number of (actual or potential) shared policy priorities between health and agriculture, based on both the agriculture sector’s mandate for production, linked to economic objectives, as well as its traditional responsibility for food (and nutrition) security. In particular, SSB taxation offers potential for revenue raising, which could be invested in subsidies and strengthening supply chains (including infrastructure for transport and storage) for healthy traditional crops. Globally, the limited investment in delivering traditional foods – particularly research and development to enhance yields and reduce post-harvest losses – has been a ‘supply side’ contributor to dietary shifts to more convenient and transportable western staples [46]. Subsidies in agriculture have tended to be for export crops, and traditional crops have been neglected [47]; particularly, traditional grains (millets), root crops, leafy green and other vegetables, pulses and fruit, which are often subject to high post-harvest losses. This would provide benefits to the agriculture sector: including reduced post-harvest losses (bringing economic benefits to farmers); improved environmental sustainability, as traditional crops are more likely to be climate-appropriate [48,49]; and improved livelihoods for small hold farmers [50], who are often more likely to produce traditional crops because of the lower technological and input requirements [49]. Directing the revenue from SSB taxation to these types of agricultural investments would support increased availability and affordability of healthy traditional foods, thus creating positive incentives for consumption and supporting both agriculture and health sector objectives.

## Discussion

With the exception of South Africa, sub-Saharan Africa remains one of the few regions in the world where dietary change associated with NCDs is at a relatively early stage, and there is potential to avert (or at least mitigate) the NCD epidemic seen in other regions. The findings of this study, high level (whole-of-government) recognition of NCDs as an important policy challenge, across all countries, is encouraging for the public health community and suggests that a policy ‘window’ exists for strengthening action on NCDs. To maximize this policy window, this study suggests that there is a need for strategic action by the health sector to shift the economic discourse, promote positive public opinion and forge links with the agriculture sector for common policy objectives to enhance the attractiveness of SSB taxation as a policy option. Political priority at the central government level is critical for the adoption of strong regulation [21].

Strong leadership across governments will be needed to counter the entrenched for profit, commercially driven, global economic incentives that are currently undermining regulation of SSBs and other unhealthy food products. Trade liberalization and export oriented economic growth have been fostering the nutrition transition for decades [41], and this dominant paradigm in the economic sectors has made food system policy change for nutrition difficult [51]. Our study suggests that the location of (multisectoral) policy responsibility for NCDs within Ministries of Health (found in 5 countries) may hamper strong regulatory action. Firstly, due to the lack of influence of the Ministry of Health over the politically strong economic sector. Secondly, the historical focus of the Ministry of Health on communicable diseases and treatment, continues to shape policy priorities. As a result, the achievement of whole-of-government objectives for NCDs and health would be better supported by developing effective country level institutional structures that enable cross-sectoral collaboration for health. Two countries (Namibia and Botswana) had NCD committees led by central Government agencies, which seemed to foster and enable future SSB taxation and offers a potential approach for strengthening early action on NCD prevention more broadly.

A key finding of this research is that SSB taxation is a politically sensitive issue, due to SSB–related industries being seen as important stakeholders for achieving government economic agendas. In the seven countries, there was an evident tension between economic sector goals, which include encouraging growth in the food industry as pivotal to economic growth, and health sector objectives to implement strong regulation (such as SSB taxes) to prevent NCDs. This finding resonates with other related research in the region, which found underlying tensions with respect to the dominant economic paradigm in economic sectors of government, which are given an explicit mandate to promote industry growth, but usually no formal mandate to consider health [51–53]. In addition, the manufacturing sector, including the food industry, is a major contributor to the economy in all the study countries, ranging from 5–10% of GDP in 2018 [54]. As a result, the food industry is a priority for the economic sector, which means that industry concerns regarding taxation have a high level of resonance with fiscal policy makers. Public health intervention that impacts the food industry would benefit from analysis that considers the potential for unintended impacts on the economy. Although excise taxation is commonly (and appropriately) used to disincentivize consumption of socially and health harmful products [55], fiscal policy makers are well aware of the potential economy-wide impacts of taxation, including distorting incentives for industry and discouraging foreign direct investment. However, current global evidence suggests that the risk of unintended consequences to the economy from non-discriminatory SSB taxes is minimal with a critical impact on healthy food choices at the population level [20]. In the future, escalating rates of NCDs will hamper economic development in the region [56]. As in other contexts, the need for revenue raising was the basis for existing taxation, which may also prove to be a point of leverage for promoting stronger SSB taxation. Across the region, achieving Univeral Health Coverage is a key goal for governments. SSB taxation as an intervention that both raises revenue and contributes to prevention and improved nutrition (thus reducing the long term burden on the health system) can play a critical role in achieving that goal.

Despite global assertions by major industry actors that they are keen to promote societal wellbeing [57,58], this study found uniformly that industry interests centred on profit maximization. In addition, industry actors engaged in direct lobbying against strong NCD prevention measures, as well as industry-led advocacy against SSB taxation on the basis of possible job losses. In South Africa, industry acted in a coordinated way to oppose the proposed SSB tax, including through direct political lobbying against taxation, discrediting scientific evidence, producing their own evidence for economic impacts, framing the primary cause of NCDs as sedentary lifestyles (or physical inactivity) and funding physical activity-based health interventions [59,60]. Similarly, data from the USA shows that the industry spent millions of dollars opposing city-based SSB taxes [61], and established industry front-groups to oppose taxation, which were portrayed as grassroots organisations that expressed the views of financially struggling families, and small businesses [62]. These findings also reflect the general approach to political influence of the food industry in Fiji, which has positioned themselves as part of the solution by sponsoring major sporting events and emphasizing the importance of their economic contribution [63].

### Strengths and limitations

This study is based on empirical research in seven countries, based on a regional protocol that was collaboratively developed by all study teams, adapted to the local context and led by researchers in-country. Given that SSA is an under-researched region with respect to NCDs, this study represents – methodologically and with respect to its findings – an important addition to the literature on NCD prevention. In addition, the explicit political economy perspective that underpinned this analysis has helped to understand the underlying reasons for the limited adoption of taxation in the region. The main limitation of the study is that not all countries were able to conduct qualitative research due to funding constraints. As a result, not all countries had access to actor opinions and perceptions of the ideas, interests, influence and institutional structures underlying the policy context. In addition, there was limited availability of data on industry activity, which hampered the analysis of corporate political activity [23].

## Conclusions

This study analysed the political economy of SSB taxation in seven countries in southern and east Africa. Five countries had existing taxes on soft drinks, primarily implemented for revenue raising purposes rather than health. SSB taxation is evidently a contested politico-economic issue, facing challenges from entrenched economic paradigms for industry-led economic growth and is actively opposed by SSB-related industries. Strategies that would enhance public health advocacy on this issue include shifting the economic discourse, including showing the economic impact of diagnosing and caring for people with NCDs. Legislating SSB taxes also motivates people to alter their consumption patterns and create new social norms in the process. Forging links with the Agriculture sector for common policy objectives should be explored. Advocating for NCD prevention to be led by a central government agency, such as the President’s office or National Planning directorates, may also increase the likelihood of the adoption of strong policy approaches. SSB taxation is one of several levers that must be used urgently across the continent to address the growing NCD epidemic that is driven by commercial interests. In the midst of the current economic challenges associated with COVID-19, SSB taxation also presents a new source of revenue with concomitant health benefits.

## Paper context

This study examined the political economy of sugar-sweetened beverage taxation in seven sub-Saharan African countries. A main finding was that sugar-sweetened beverage taxation is a sensitive politico-economic issue. Strategic advocacy by the health sector will be required to overcome sugar-sweetened beverage industry opposition, which is often predicated on their contribution to core economic policy objectives. Key strategies include, shifting the economic discourse to recognize public health objectives, supporting cultural change, and forging stronger links with the agriculture sector.
